# Trends in surgical approach and pediatric pulmonology follow-up for operated congenital thoracic malformations

**DOI:** 10.3389/fped.2026.1835493

**Published:** 2026-08-31

**Authors:** Çelebi Yıldırım, Gökçen Dilşa Tuğcu, Satı Özkan Tabakçı, Işıl Bilgiç, Meltem Kürtül Çakar, Gamze Akça Dinç, Ayyüce Aktemur Ünlü, Hande Yetişgin, Ayça Laçin Tekin, İrem Akbaş, Süleyman Arif Bostancı, Sanem Eryılmaz Polat, Dilber Ademhan Tural, Müjdem Nur Azılı, Güzin Cinel

**Affiliations:** 1 Department of Pediatric Pulmonology, Ministry of Health, Ankara Bilkent City Hospital, Ankara, Türkiye; 2 Department of Pediatric Surgery, Ministry of Health, Ankara Bilkent City Hospital, Ankara, Türkiye; 3 Department of Pediatric Pulmonology, Faculty of Medicine, Ankara Yıldırım Beyazıt University, Ankara, Türkiye

**Keywords:** antenatal diagnosis, anthropometric measures, congenital pulmonary airway malformation, congenital thoracic malformation, long-term follow-up, mucinous adenocarcinoma, spirometry, video-assisted thoracoscopic surgery

## Abstract

**Introduction:**

Congenital thoracic malformations (CTM) including congenital pulmonary airway malformation (CPAM), pulmonary sequestration (PS), bronchogenic cysts (BC), congenital lobar overinflation (CLO), and bronchial atresia (BA) are increasingly detected antenatally. Resection is generally undertaken in symptomatic patients, whereas the management of asymptomatic lesions remains controversial, with no consensus on whether or when to operate. We compared children who underwent surgery for CTMs after antenatal versus postnatal diagnosis.

**Methods:**

Pediatric patients who underwent resection of histopathologically confirmed CTM at a single tertiary center between August 2019 and December 2025, with at least 6 months' follow-up, were retrospectively evaluated for surgical approach [thoracotomy/video-assisted thoracoscopic surgery (VATS)], length of hospitalization, age at operation, and clinical, anthropometric, and spirometric outcomes at the last visit.

**Results:**

Fifty-six patients were included [29 (51.80%) male; median age at final visit 54.00 months]. Antenatal diagnosis occurred in 34 (60.70%), and CPAM was the most frequent lesion (50.00%). Preoperative radiology and postoperative histopathology were concordant in 80.40%. Median age at surgery was numerically lower in prenatally diagnosed patients, but the difference was not statistically significant (11.00 vs. 33.50 months, *p* = 0.087). VATS was performed in 43 (76.80%) and thoracotomy in 13 (23.20%). During follow-up, scoliosis occurred in 10 (17.90%) and pectus deformity in 8 (14.30%). Spirometry, available in 15 patients (26.70%), was within near-normal ranges (FVC z-score −0.77; FEV_1_ z-score −0.84). Median weight and height z-scores were −0.15 and −0.01.

**Conclusion:**

In this single-center retrospective cohort, somatic growth after resection of CTM was generally normal and lung function largely preserved, with infrequent chronic respiratory morbidity. No statistically significant differences were detected between antenatally and postnatally diagnosed patients, although the study was underpowered to exclude smaller differences and spirometry was available in only a subset of children. These observations are hypothesis-generating and support comprehensive, family-centered long-term follow-up.

## Introduction

Congenital thoracic malformations (CTMs) include anomalies of the pulmonary parenchyma and airways such as congenital pulmonary airway malformation (CPAM), pulmonary sequestration (PS), bronchogenic cysts (BC), congenital lobar overinflation (CLO), and bronchial atresia (BA) (1). CTMs are uncommon, with an estimated incidence of 1 in 2,500–10,000 live births (1, 2). CPAM is the most common subtype, with hybrid lesions (comprising features of multiple types) observed less frequently (3).

Advances in fetal ultrasonography (USG) and magnetic resonance imaging (MRI) have significantly increased antenatal detection rates, enabling the early diagnosis of CTMs. However, postnatal imaging and histopathological correlation remain essential for definitive diagnoses (1, 3). Computed tomography angiography (CTA) is a valuable postnatal imaging technique for evaluating lesion type and vascularity (1).

Resection is generally undertaken in symptomatic patients. In contrast, the management of asymptomatic lesions remains controversial: there is no definite recommendation as to whether or when asymptomatic CTMs should be resected, and reported practice varies considerably between centers. Despite the growing body of data, a critical information gap remains: there is no consensus on pediatric pulmonology follow-up for patients who have undergone surgery for CTMs (4). A multidisciplinary consensus on the optimal timing of surgery, length of follow-up, and standard monitoring protocols for this patient population is required.

This study aimed to assess the demographic, clinical, anthropometric, and spirometric characteristics of patients who underwent surgical treatment for CTMs and to compare outcomes between those diagnosed in the prenatal and postnatal periods, beyond the scope of pediatric pulmonology. The characteristics of patients who underwent video-assisted thoracoscopic surgery (VATS) and those who underwent thoracotomy were also compared. Long-term postoperative outcomes were evaluated by analyzing anthropometric measures, the frequency of respiratory symptoms (recurrent/refractory pneumonia, asthma, chronic cough, and wheezing), and spirometry tests during follow-up at our pediatric pulmonology outpatient clinic.

## Materials and methods

### Study design and patient population

This retrospective cohort study included pediatric patients who underwent surgical resection of CTMs at a single tertiary center between August 2019 and December 2025.

The inclusion criteria were as follows:

1. Surgery due to CTMs based on clinical and radiological findings, with diagnosis confirmed by histopathological examination;
2. A follow-up period of at least 6 months; and
3. The availability of complete medical records, including prenatal imaging reports, surgical notes, and follow-up data.

Patients with chromosomal anomalies, insufficient follow-up data, or lack of histopathological confirmation of the CTM were excluded.

Patients were evaluated based on the time of diagnosis (prenatal or postnatal), the age at surgery (months), and the surgical method (thoracotomy or VATS).

This study was approved by the Ankara Bilkent City Hospital Ethics Committee (TABED 1-25-1548) and conducted in accordance with the Declaration of Helsinki.

### Data collection and monitoring parameters

All data were collected retrospectively from electronic medical records and imaging archives. The key demographic and clinical characteristics evaluated are summarized as follows:

- Prenatal data: includes patients diagnosed with CTMs prenatally through fetal USG/MRI.
- Postnatal data: comprises patients diagnosed and treated postnatally.
- Patients were analyzed according to the surgical method used (VATS vs. thoracotomy). Postoperative hospitalization length was recorded and analyzed in days.
- Patient clinical and demographic data were collected. Long-term follow-up was conducted in pediatric pulmonology and surgery clinics, including anthropometric assessments, evaluation of thoracic and spinal deformities, and documentation of long-term postoperative respiratory symptoms such as pneumonia episodes, recurrence of asthma or reactive airway disease, and chronic cough/wheezing.
- Anthropometric data, including body weight and recumbent length (for patients aged < 2 years) or standing height (for patients aged ≥ 2 years), were obtained at the final visit. Age- and sex-specific percentiles and z-scores (< 2 years) and (≥ 2 years) were determined using the Centers for Disease Control and Prevention 2000 growth reference charts.
- Radiological findings: USG and MRI findings of patients diagnosed prenatally were reviewed where available. CTA and chest radiography (CXR) findings were also reviewed, along with follow-up if necessary, including the lesion size, anatomical location, and vascularity.
- Pulmonary function tests: Spirometry was performed on patients aged ≥ 6 years who were able to cooperate. All spirometric procedures were performed in accordance with the American Thoracic Society and European Respiratory Society (ATS/ERS) standards by the same certified technicians (5). Spirometry indices were analyzed using the reference values of Quanjer et al. (6). Spirometry curves were reevaluated by a single senior pediatric pulmonologist following the recently published update of the ATS/ERS standardization of spirometry (7).
- Histopathology: Final histopathological diagnosis after surgical resection (e.g., CPAM, PS, BC, CLO, hybrid lesion) was made by a pediatric pathologist.

### Statistical analysis

Statistical analyses were conducted using IBM SPSS Statistics version 26. Categorical variables were summarized using frequencies and percentages; numerical variables were summarized using medians, interquartile ranges and percentiles. The distribution of continuous variables was assessed using Q–Q plots, P–P plots, histograms, and formal normality tests, including the Shapiro–Wilk and Kolmogorov–Smirnov tests. The Mann–Whitney U test was applied for two-group analyses. Associations between categorical variables were examined using the Pearson's chi-square test or Fisher's exact test. Correlations between non-normally distributed continuous variables were assessed using Spearman's rank correlation coefficient. Because patients were not randomly allocated to the two surgical approaches and a statistically significant difference in age at operation was observed between the groups, the effect of surgical approach on postoperative weight z-score was assessed using analysis of covariance (ANCOVA; general linear model, univariate procedure, Type III sum of squares). Surgical approach (thoracotomy vs. VATS) was entered as a fixed factor, with VATS as the reference category, and age at operation and duration of follow-up were entered as covariates. These covariates were selected *a priori* because they differed between the groups and were considered clinically plausible determinants of postoperative growth. Adjusted (estimated marginal) means with their standard errors were computed for each group. The assumptions of ANCOVA were verified before interpretation: normality of the model residuals (Shapiro–Wilk test and Q–Q plots), homogeneity of error variances (Levene's test), linearity of the relationship between each covariate and the dependent variable (inspection of scatterplots), and homogeneity of regression slopes, the last of which was tested by adding surgical approach   ×   covariate interaction terms to the model. The interaction terms were non-significant, confirming that the assumption was met; these terms were therefore removed from the final model. Results are reported as unstandardized regression coefficients (B) with 95% confidence intervals. Effect sizes are expressed as partial eta squared (*η*^2^ₚ) and interpreted according to Cohen's criteria as small (0.01), medium (0.06), or large (0.14). A two-tailed *p*-value of < 0.05 was considered statistically significant. Due to the retrospective design, the sample size was not determined by an *a priori* power calculation. *Post hoc* power based on observed effect sizes was not computed, as observed power is a monotonic function of the *p*-value and provides no information beyond the reported significance level. Instead, statistical power was estimated for conventionally defined effect sizes (Cohen's d = 0.5 and d = 0.8), and sensitivity analyses were conducted to determine the minimum detectable effect size given the available sample. A two-tailed *α* of 0.05 was assumed for all calculations, which were performed using G*Power 3.1 (8).

## Results

### Patient characteristics

**Figure 1:**
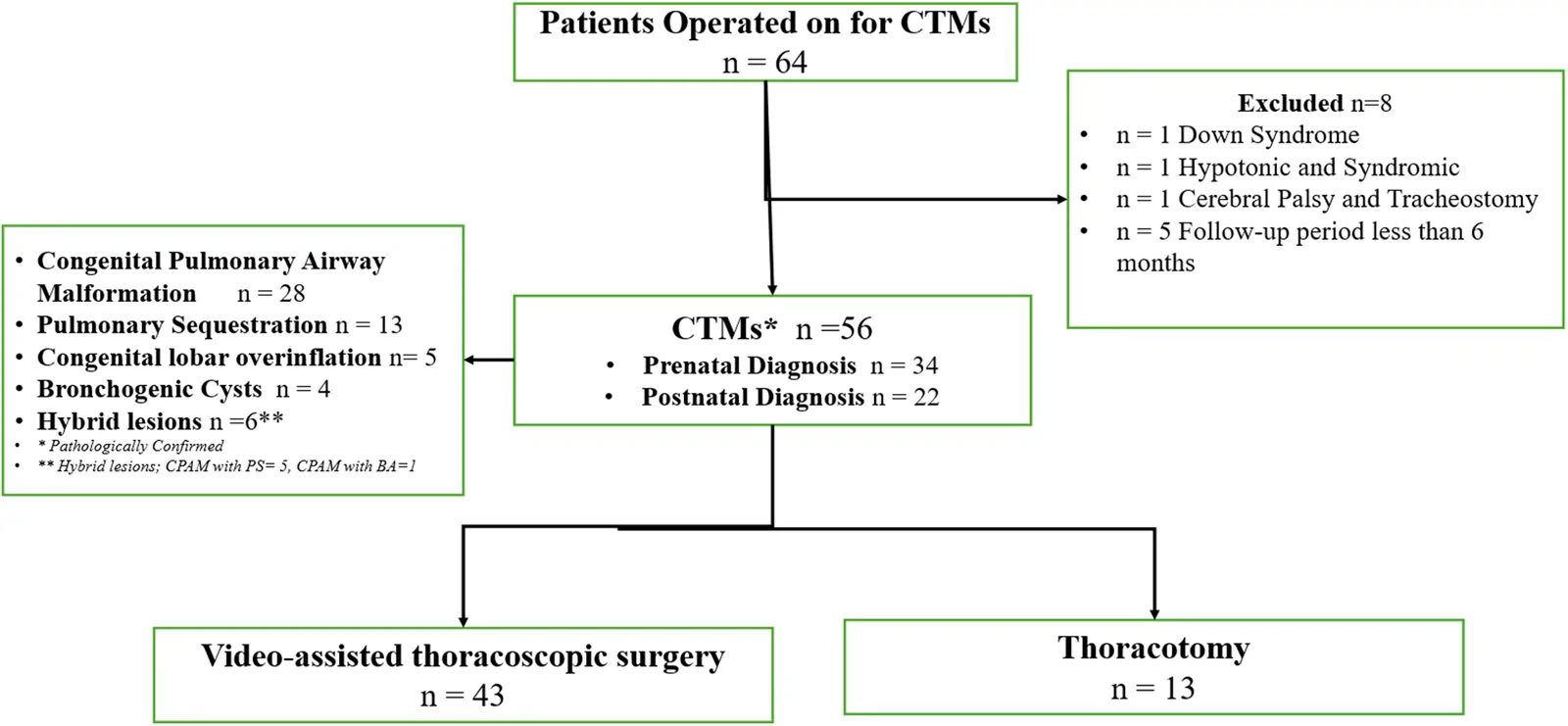
Flow chart of the study.

This study included 56 patients who underwent surgery for CTMs (Figure 1). A total of 29 patients (51.80%) were male, and the median age at the final follow-up visit was 54.00 months (Q1–Q3: 25.00–90.00). The median age at surgery was 12.50 months (Q1–Q3: 4.00–46.20), the postoperative median length of hospitalization was 11.00 days (Q1–Q3: 8.00–21.00), and the median follow-up period was 20.50 months (Q1–Q3: 9.00–50.50).

Diagnosis was made prenatally in 34 patients (60.70%), with 22 (39.30%) diagnosed postnatally. The median age of diagnosis in the postnatal period was 33.50 months (Q1–Q3: 3.75–100.50). VATS was performed in 43 patients (76.80%) and thoracotomy in 13 patients (23.20%). All demographic data are summarized in Table 1. Details on the study population are provided in Figure 1.

**Table 1:** The demographic and clinical characteristics of the cohort.

Characteristics		Total cohort (*n* = 56)
Age, Median, Q1–Q3, (months)		54.00 (25.0 to 90.0)
Gender, Male/Female, n (%)		29 (51.80)/ 27 (48.20)
Diagnosed antenatally n (%)		34 (60.70)
CXR at Diagnosis n (%)	**Normal**	7 (12.50)
	**Mediastinal Shift**	8 (14.30)
	**Cystic lesions**	25 (44.60)
	**Hyperinflation**	11 (19.60)
Chest Computed Tomography (*n* = 54) n (%)	**Multiple cysts**	23 (42.60)
	**Lesion supplied by an aberrant artery**	12 (22.20)
	**Cavity, cystic lesion**	7 (13.00)
	**Multiple cysts supplied by aberrant arteries**	5 (9.300)
	Others*	7 (13.00)
Age at time of operation median, Q1–Q3, (months)		12.50 (4.00 to 46.20)
Surgery, n (%)		
✓ VATS n (%)		43 (76.80)
✓ Thoracotomy n (%)		13 (23.20)
Length of hospitalization after surgery, median, Q1–Q3, (days)		11.00 (8.00 to 21.00)
Follow-up period median, Q1–Q3, (months)		20.50 (9.00 to 50.50)
✓ Congenital Thoracic Malformation* (n, %)		
✓ Congenital Pulmonary Airway Malformation n (%)		28 (50.00)
✓ Pulmonary Sequestration n (%)		13 (23.20)
✓ Congenital lobar overinflation n (%)		5 (8.90)
✓ Bronchogenic Cysts n (%)		4 (7.10)
✓ Hybrid lesions** n (%)		6 (10.70)
Malignancy		2 (3.50)

* Pathologically Confirmed, ** Hybrid lesions; 6 patients: CPAM with PS = 5, CPAM with BA = 1, CPAM, congenital pulmonary airway malformation; PS, pulmonary sequestration; BC, bronchogenic cysts; CLO, congenital lobar overinflation; BA, bronchial atresia; CXR, chest radiography; VATS, video-assisted thoracoscopic surgery.

At diagnosis, the most frequent chest radiography findings were cystic lesions in 25 patients (44.60%), hyperinflation in 11 (19.60%), and mediastinal shift in 8 (14.30%). Normal radiographs were observed in 7 (12.50%) patients (Table 1). In total, 54 (96.40%) patients were imaged using computed tomography (CT); the most frequent finding was multiple cysts, observed in 23 patients (42.60%). Lesions supplied by an aberrant artery were detected in 12 patients (22.20%), while cavity or cystic lesions were found in 7 patients (13.00%). Multiple cysts supplied by aberrant arteries were relatively infrequent, occurring in 5 patients (9.30%) (Table 1).

### Prenatal/postnatal diagnosis

According to the final clinical evaluation performed to determine postnatal surgical indication in the prenatally diagnosed group, a total of 34 cases were included. Of these, 22 (64.70%) were asymptomatic and electively operated, 6 (17.60%) had acute respiratory failure in the early postnatal period, 4 (11.80%) had lower respiratory tract infection, and 2 (5.90%) had insufficient weight gain (weight z-score below −2). In asymptomatic cases, the median time to operation was 11.50 months (Q1–Q3: 8.00–22.00). This period was 4.50 months (Q1–Q3: 1.00–24.00) in symptomatic cases (U = 84.5, Z = −1.718, *p* = 0.087). The postnatal group included 22 cases. Of these, 10 (45.50%) were asymptomatic and incidentally diagnosed, 7 (31.80%) had recurrent/refractory lower respiratory tract infections, and 5 (22.70%) had acute respiratory failure. Four patients were diagnosed in the early postnatal period and one patient at 15 months.

**Figure 2:**
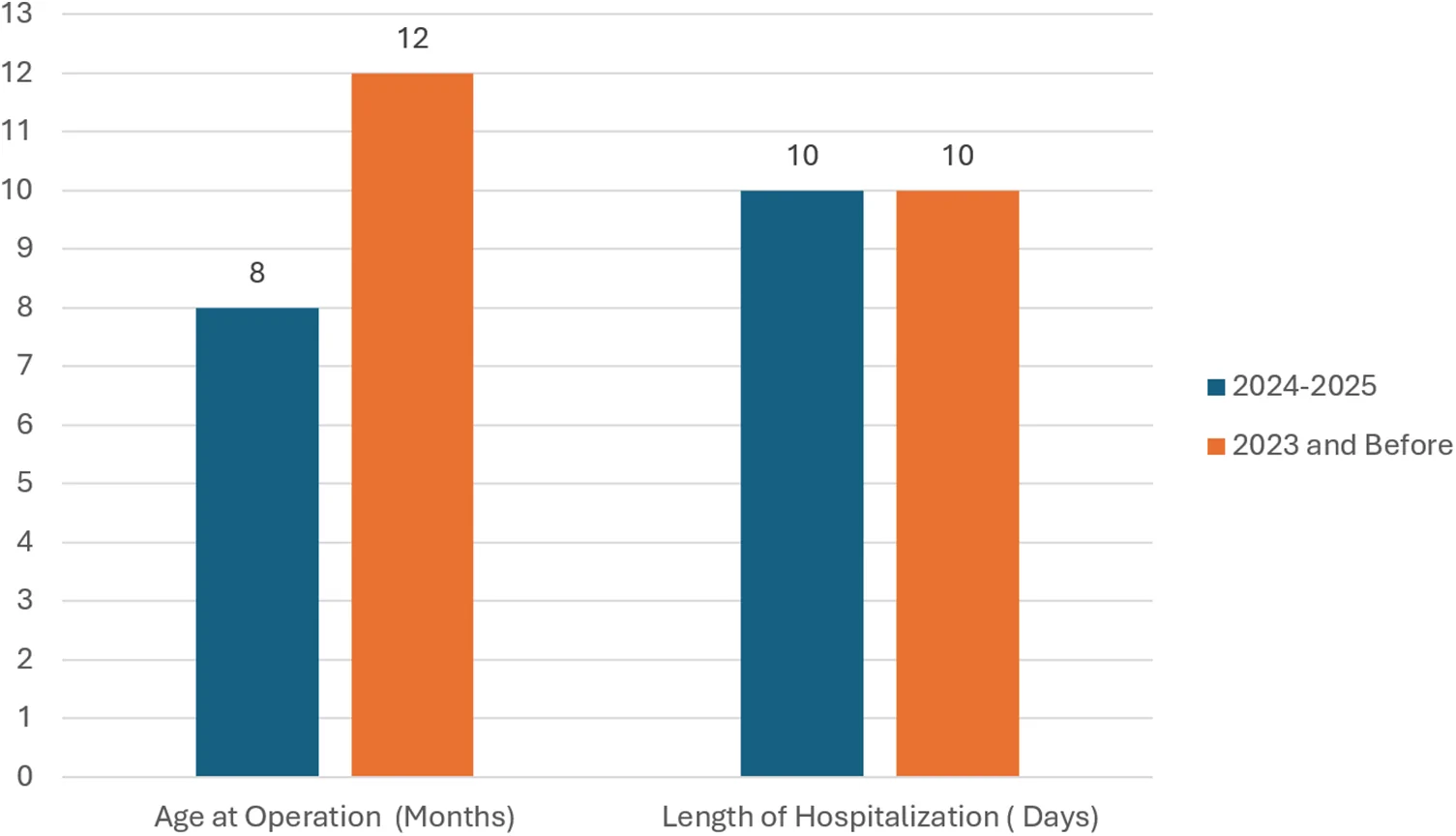
Patient Age at operation and length of hospitalization in 2023-before and 2024–2025.

The median age of the prenatally diagnosed patients was 11.00 months at operation (Q1–Q3: 4.75 to 22.00). This value was 33.50 months (Q1–Q3: 4.00–100.50) in postnatally diagnosed patients; however, the difference was not statistically significant (U = 272.5, Z = −1.709, *p* = 0.087). No statistically significant difference in the postoperative length of hospitalization was found between patients diagnosed prenatally (median 10.00 days, Q1–Q3: 8.00–18.00) and postnatally (median 14.0 days, Q1–Q3: 8.50–29.0), although prenatally diagnosed patients were hospitalized for fewer days (*p* = 0.210) (Figure 2).

### Surgery type and length of hospitalization

The median age at surgery was 4.00 months (Q1–Q3: 1.00–10.50) in 13 (23.20%) patients who underwent thoracotomy. This value was 19.00 months (Q1–Q3: 6.00–67.00) in the VATS group (*n* = 43), with a statistically significant difference (*p* = 0.003). The median length of hospitalization was 14.00 days (Q1–Q3: 10.00–58.00) in the thoracotomy group and 10.00 days (Q1–Q3: 7.00–18.00) in the VATS group; however, this difference was nonsignificant (*p* = 0.106). It was not possible to obtain retrospective data on operation duration.

### Trends over the years

Patient age at operation, length of hospitalization, and type of surgery were evaluated for the years 2023-before and 2024–2025. The median age at operation was 12.00 months (Q1–Q3, 6.00–26.00) in 2019–2023, and 8.00 months (Q1–Q3: 5.00–11.00) in 2024–2025 (*p* = 0.615). The median length of hospitalization was 10.00 days in both 2023-before and 2024–2025 (Q1–Q3: 8.00–21.00; Q1–Q3: 7.00–11.00; *p* = 0.179) (Figure 2).

**Figure 3:**
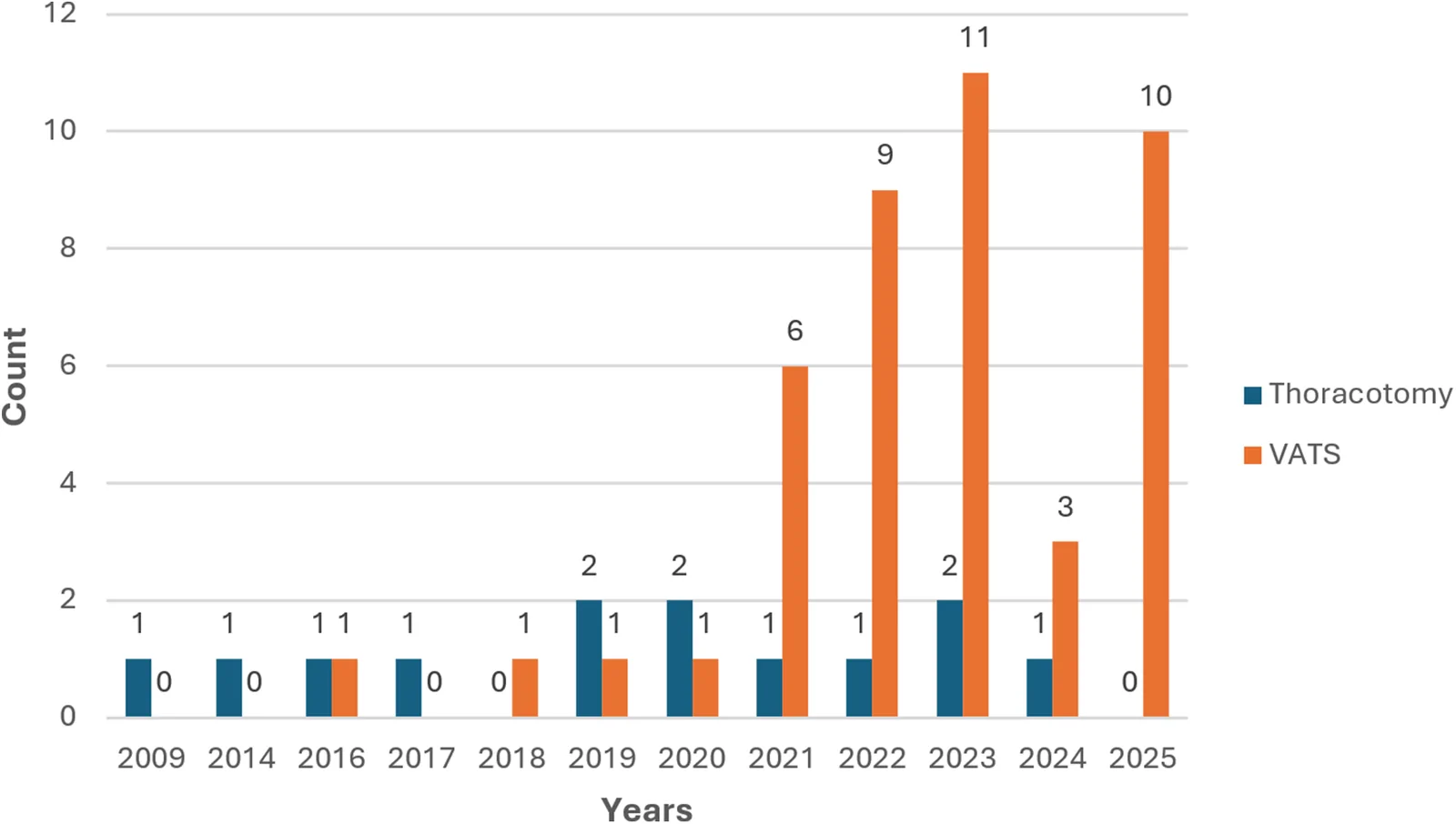
Surgical procedures [thoracotomy vs. Video-Assisted Thoracoscopic Surgery (VATS)] Performed.

Most surgeries performed in the last two years were VATS; thoracotomy was preferred in only one case (VATS: 13; thoracotomy: 1) (Figure 3). Of the 13 patients who underwent thoracotomy, 8 (61.50%) began with primary thoracotomy. A total of 5 patients (38.50%) required conversion to thoracotomy from VATS during the intraoperative period.

### Histopathological confirmation

The pathological diagnoses of the cohort were as follows: CPAM in 28 (50.00%) patients, PS in 13 (23.20%) patients, hybrid lesions in 6 patients (10.70%) (CPAM + PS = 5; CPAM + BA = 1), CLO in 5 patients (8.90%), and BC in 4 patients (7.10%). Examining the histopathological subtype distribution, the most common variants were CPAM type 2 (*n* = 14; 50.00%), CPAM type 1 (*n* = 9; 32.10%), and CPAM type 4 (*n* = 1; 3.60%). Detailed diagnostic subtype data were unavailable for the remaining 2 cases (7.10%). Mucinous adenocarcinoma arising from CPAM type 1 was identified in 2 cases (7.10%).

The preoperative radiologic diagnoses matched the postoperative pathologic diagnoses in 45 (80.4%) patients. The characteristics of patients with differing preoperative radiological and postoperative histopathological diagnoses are provided in Table 2.

**Table 2:** Histopathological diagnosis of patients inconsistent with radiology.

Number of patients	Radiological Diagnosis	Histopathological Diagnosis
1	Lung Abscess	Bronchogenic cyst
1	CPAM type 1	Mucinous Adenocarcinoma and CPAM type 1
2	Hydatid Cyst	CPAM type 1
1	CPAM type 3	CPAM type 1
1	Hybrid lesion (PS with BA)	PS
2	CPAM	PS
1	CPAM	Bronchogenic cyst
1	Diaphragmatic Hernia	PS
1	Pleuropulmonary Blastoma and CPAM type 4	Mucinous Adenocarcinoma and CPAM type 1

CPAM, congenital pulmonary airway malformation; PS, pulmonary sequestration; BC, bronchogenic cysts; BA, bronchial atresia.

### Respiratory symptoms, spirometry, and anthropometric measures

The median weight z-score was −0.15 (Q1–Q3: −0.57–0.50), and the median height z-score was −0.01 (Q1–Q3: −0.63–0.56), indicating that overall growth parameters were within normal reference ranges at the final follow-up visit (Table 3). There were no statistically significant differences between prenatally and postnatally diagnosed patients in the duration of follow-up and height/weight z-scores at the final visit (Table 4).

**Table 3:** Long-Term follow-Up and comorbidities.

Variables		Total *n* = 56
Weight z-score at the last follow-up visit, median, Q1–Q3		−0.15 (−0.57 to 0.50)
Height z-score at the last follow-up visit, median, Q1–Q3		−0.01 (−0.63 to 0.56)
Spirometry, (*n* = 15)	**FVC** (%) median, Q1–Q3	78.00 (60.00 to 94.00)
	**FVC z-score** median, Q1–Q3	−0.77 (−1.98 to −0.31)
	**FEV_1_** (%) median, Q1–Q3	81.00 (64.00 to 100.00)
	**FEV_1_ z-score** median, Q1–Q3	−0.84 (−2.43 to −0.02)
	**FEV_1_/FVC** (%) median, Q1–Q3	96.00 (92.00 to 107.00)
	**FEF_25−75_** (%) median, Q1–Q3	81.00 (68.00 to 107.00)
	**FEF_25−75_ z-score** median, Q1–Q3	−0.76 (−0.84 to 0.30)
Pectus excavatum/carinatum, (n %)		8 (14.30)
Recurrent/Refractory Pneumonia, (n %)		5 (8.90)
Scoliosis (n %)		10 (17.90)
Wheezing/Stridor (n %)		5 (8.90)
Asthma (n %)		9 (16.10)

FVC, forced vital capacity; FVC z-score, z-score of forced vital capacity; FEV₁, forced expiratory volume in the first second; FEV₁ z-score, z-score of forced expiratory volume in the first second; FEV₁/FVC, ratio of forced expiratory volume in the first second to forced vital capacity; FEF₂₅–₇₅, forced expiratory flow between 25% and 75% of forced vital capacity; FEF₂₅–₇₅ z-score, z-score of forced expiratory flow between 25% and 75% of forced vital capacity.

**Table 4:** Association between diagnosis time and surgery type with clinical variables.

Variables		Prenatal diagnosis (*n* = 34)	Postnatal diagnosis (*n* = 22)	p
Age of Operation (months), median Q1–Q3		11.00 (4.75 to 22.00)	33.50 (4.00 to 100.50)	0.087
Surgery,	**Thoracoscopic (VATS)** n (%)	29 (85.30)	14 (63.60)	0.061^+^
	**Thoracotomy** n (%)	5 (14.70)	8 (36.40)	
Length of hospitalization, median Q1–Q3		10.00 (8.00 to 18.00)	14.00 (8.50 to 29.00)	0.210
Follow-up duration (months) median Q1–Q3		24.00 (8.75 to 39.25)	19.50 (9.00 to 60.50)	0.626
Weight z-score at the last follow-up visit median Q1–Q3		−0.01 (−0.77 to 1.10)	−0.25 (−0.52 to 0.21)	0.632
Height z-score at the last follow-up visit median Q1–Q3		−0.01 (−0.68 to 0.62)	−0.03 (−0.64 to 0.32)	0.887
Spirometry Test	**FVC (%)** median Q1–Q3	78.00 (64.50 to 83.00)	88.00 (59.75 to 118.50)	0.371
	**FVC z-score** median Q1–Q3	−1.54 (−2.12 to −1.00)	−0.52 (−1.40 to 1.21)	0.099
	**FEV_1_ (%)** median Q1–Q3	79.00 (70.00 to 87.00)	87.00 (61.50 to 108.00)	0.440
	**FEV_1_ z-Score** median Q1–Q3	−1.49 (−2.29 to −0.80)	−0.65 (−2.64 to 0.82)	0.440
	**FEV_1_/FVC (%)** median Q1–Q3	103.00 (99.00 to 108.00)	92.50 (87.75 to 102.00)	0.075
	**FEF_25−75_ (%)** median Q1–Q3	81.00 (73.50 to 108.00)	80.00 (61.25 to 103.00)	0.594
	**FEF_25−75_ z-score** median Q1–Q3	−0.76 (−1.09 to 0.27)	−0.37 (−1.08 to 0.47)	0.953
Pectus excavatum/carinatum n (%)		4 (11.80)	4 (18.20)	0.698*
Recurrent/refractory pneumonia n (%)		3 (8.80)	2 (9.10)	1.000*
Scoliosis n (%)		4 (11.80)	6 (27.30)	0.167*
Wheezing/Stridor n (%)		3 (13.60)	2 (3.60)	0.371*
Asthma n (%)		6 (17.60)	3 (13.60)	1.000*

		VATS (*n* = 43)	Thoracotomy (*n* = 13)	p
Diagnosis	**Prenatal** n %	29 (67.40)	5 (38.50)	0.061^+^
	**Postnatal** n %	14 (32.60)	8 (61.50)	
Age of Operation (months) median Q1–Q3		19.00 (6.00 to 67.00)	4.00 (1.00 to 10.50)	**0.003**
Length of hospitalization median Q1–Q3		10.00 (7.00 to 18.00)	14.00 (10.00 to 58.00)	0.106
Follow-up duration (months) median Q1–Q3		13.00 (8.00 to 32.00)	60.00 (17.00 to 87.00)	**0.001**
Weight z-score at the last follow-up visit median Q1–Q3		0.10 (−0.50 to 1.11)	−0.50 (−1.39 to −0.13)	**0.004**
Height z-score at the last follow-up visit median Q1–Q3		0.05 (−0.50 to 0.85)	−0.20 (−0.77 to 0.01)	0.068
Spirometry Test	**FVC(%)** median Q1–Q3	88.0 (72.0 to 108.0)	67.50 (55.0 to 84.0)	0.104
	**FVC z-score** median Q1–Q3	−0.65 (−1.98 to 1.19)	−0.99 (−2.00 to −0.54)	0.571
	**FEV_1_ (%)** median Q1–Q3	89.00 (76.00 to 108.00)	72.00 (54.00 to 84.00)	0.138
	**FEV_1_ z-score** median Q1–Q3	−0.66 (−2.16 to 0.80)	−1.67 (−2.90 to −0.69)	0.226
	**FEV_1_/FVC** median Q1–Q3	96.00 (92.00 to 107.00)	97.00 (76.00 to 110.00)	1.000
	**FEF_25−75_ (%)** median Q1–Q3	98.00 (74.00 to 109.00)	68.00 (47.00 to 81.00)	0.056
	**FEF_25−75_ z-score** median Q1–Q3	0.13 (−0.84 to 0.33)	−0.82 (−1.68 to −0.15)	0.177
Pectus excavatum/carinatum n %		5 (11.60)	3 (23.10)	0.370*
Recurrent/refractory Pneumonia n %		3 (7.00)	2 (15.40)	0.580*
Scoliosis n %		7 (16.30)	3 (23.10)	0.682*
Wheezing/Stridor n %		4 (9.30)	1 (7.70)	1.000*
Asthma n %		6 (14.00)	3 (23.10)	0.419*

+*P* values were calculated using Pearson chi-square test. *P values were calculated using Fisher's exact test. FVC, forced vital capacity; FVC z-score, z-score of forced vital capacity; FEV₁, forced expiratory volume in the first second; FEV₁ z-score, z-score of forced expiratory volume in the first second; FEV₁/FVC, ratio of forced expiratory volume in the first second to forced vital capacity; FEF₂₅–₇₅, forced expiratory flow between 25% and 75% of forced vital capacity; FEF₂₅–₇₅ z-score, z-score of forced expiratory flow between 25% and 75% of forced vital capacity; VATS, video-assisted thoracoscopic surgery.

The median weight z-score at the final visit was −0.50 (Q1–Q3: −1.39 to −0.13) in the thoracotomy group and 0.10 (Q1–Q3: −0.50–1.11) in the VATS group. This difference was statistically significant (*p* = 0.004). The median height z-score at the final visit was −0.20 (Q1–Q3: −0.77–0.01) in the thoracotomy group and 0.05 (Q1–Q3: −0.50–0.85) in the VATS group; however, this difference was nonsignificant (*p* = 0.068). In the long-term follow-up, no statistically significant differences were found between scoliosis and pectus deformity, frequency of recurrent pneumonia, or spirometric parameters by operation type (Table 4).

After adjusting for age at surgery and follow-up duration, surgical approach remained an independent predictor of weight z-score, with the thoracotomy group scoring 1.06 SD lower than the VATS group (95% CI −1.76 to −0.36; *p* = 0.004; partial *η*^2^ = 0.150). Although the two groups differed in median age at operation (4.00 vs. 19.00 months), age was not a significant covariate (*p* = 0.363), and the adjusted difference was marginally larger than the unadjusted one, indicating that this imbalance did not account for the observed effect. Residual confounding by underlying disease severity cannot be excluded, as thoracotomy was preferentially performed in younger, more symptomatic patients.

Concerning clinical parameters, it was observed that patient age at operation was not related to the length of hospital stay (rho = −0.222, *p* = 0.113), weight z-scores (rho = 0.064, *p* = 0.638), or height z-scores (rho = −0.010, *p* = 0.941). Moreover, no significant associations were observed between the length of hospital stay and weight (rho = −0.082, *p* = 0.563) and height (rho = 0.047, *p* = 0.743) z-scores.

Scoliosis was the most common comorbidity documented during follow-up, affecting 10 patients (17.90%). Asthma was diagnosed in 9 patients (16.10%) and pectus deformity (pectus excavatum/carinatum) in 8 patients (14.30%). Recurrent or refractory pneumonia and wheezing/stridor were reported in 5 patients (8.90%). Spirometric assessment was performed in 15 (26.70%) patients, with all parameters within near-normal ranges. The spirometry test parameters [median (Q1–Q3)] were as follows: FVC 78% (60.00–94.00), FVC z-score −0.77 (−1.98 to −0.31); FEV_1_ 81.00% (64.00–100.00), FEV_1_ z-score −0.84 (−2.43 to −0.02), and FEV_1_/FVC ratio 96.00% (92.00–107.00) (Table 3). The median age of the 15 patients who underwent spirometry was 90.00 months (Q1–Q3: 72.00–129.00 months).

The median FVC was 78.00% (Q1–Q3: 64.50–83.00) in the prenatal group and 88.00% (Q1–Q3: 59.75–118.50) in the postnatal group (*p* = 0.371). Although the prenatal group had lower median FVC z-scores of −1.54 [Q1–Q3: −2.12 to −1.00] and −0.52 [Q1–Q3: −1.40–1.21], this difference was nonsignificant (*p* = 0.099). Similarly, median FEV₁ values were 79.00% (Q1–Q3: 70.00–87.00) in the prenatal group and 87.00% (Q1–Q3: 61.50–108.00) in the postnatal group (*p* = 0.440). FEV₁ z-scores were comparable (−1.49 vs. −0.65, *p* = 0.440). The FEV₁/FVC ratio was higher in the prenatal group (103.00%; Q1–Q3: 99.00–108.00) than the postnatal group (92.50%; Q1–Q3: 87.75–102.00); however, this difference was not statistically significant (*p* = 0.075) (Table 4).

Comparing the spirometry results of the VATS and thoracotomy groups, higher values were observed in favor of VATS in most examined respiratory function parameters; however, no statistically significant difference was found between the groups. The FVC value was 88.00% (Q1–Q3: 72.00–108.00) in the VATS group and 67.50% (Q1–Q3: 55.00–84.00) in the thoracotomy group (*p* = 0.104); the FVC z-scores were −0.65 (Q1–Q3: −1.98–1.19) and −0.99 (Q1–Q3: −2.00 to −0.54), respectively (*p* = 0.571). Similarly, the FEV_1_ value was 89.00% (Q1–Q3: 76.00–108.00) in the VATS group and 72.00% (Q1–Q3: 54.00–84.00) in the thoracotomy group (*p* = 0.138); the FEV_1_ z-scores were −0.66 (Q1–Q3: −2.16–0.80) and −1.67 (Q1–Q3: −2.90 to −0.69), respectively (*p* = 0.226). A comparison of the spirometry outcomes between the VATS and thoracotomy groups is provided in Table 4.

### Malignant transformation: Two illustrative cases

#### Case 1

A newborn diagnosed with congenital pulmonary airway malformation (CPAM) prenatally underwent surgery for respiratory distress after birth. Histopathological examination of the lobectomy specimen revealed mucinous adenocarcinoma arising in CPAM type 1, and molecular analysis showed a positive KRAS mutation. Surgical margins were negative, and no residual lesions were observed. Therefore, adjuvant chemotherapy or targeted therapy does not need to be given by pediatric oncology. The patient is followed clinically every three months and with annual chest MRI. The patient, currently 33 months old, has growth and development consistent with age and has no respiratory symptoms.

#### Case 2

A 15-year-old patient underwent surgery after a thoracic computed tomography (CT) scan for hemoptysis and a preliminary diagnosis of CPAM. Histopathological evaluation of the resection specimen revealed mucinous adenocarcinoma arising from CPAM type 1; the KRAS mutation was negative. Following detection of residual disease during postoperative evaluation, chemotherapy (cisplatin, vinorelbine) and targeted therapy (bevacizumab) were administered by pediatric oncology. No disease recurrence was observed during post-treatment follow-up. The patient has been transferred to the adult medical oncology and pulmonology departments, and follow-up is being continued by these units. The patient is currently 20 years old without any complications observed. The clinical, histopathological, and molecular findings, as well as the treatment and follow-up characteristics of both cases, are summarized in Table 5.

**Table 5:** Clinical and pathological features of the two cases.

Feature	Case 1	Case 2
Age at diagnosis	Neonate (antenatally diagnosed)	15 years
Presenting feature	Respiratory distress (neonatal)	Hemoptysis; suspected CPAM on CT
Histopathology	Mucinous adenocarcinoma arising in CPAM type 1	Mucinous adenocarcinoma arising in CPAM type 1
Location	Left lower lobe (Op: lobectomy)	Left lower lobe (Op: lobectomy)
KRAS positivity in lobectomy materials.	Positive	Negative
Resection margins	Negative	Positive (Residual Disease)
Adjuvant therapy	Not given	Given
Preoperative imaging	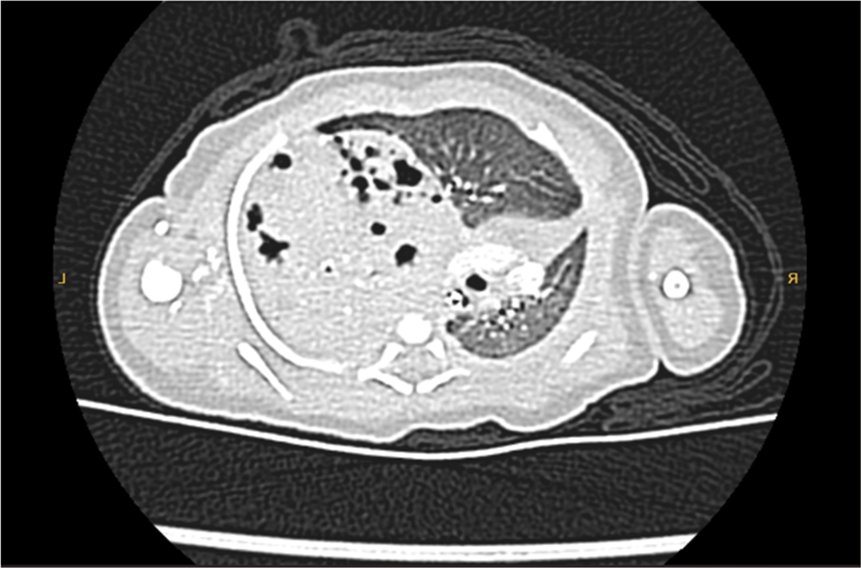	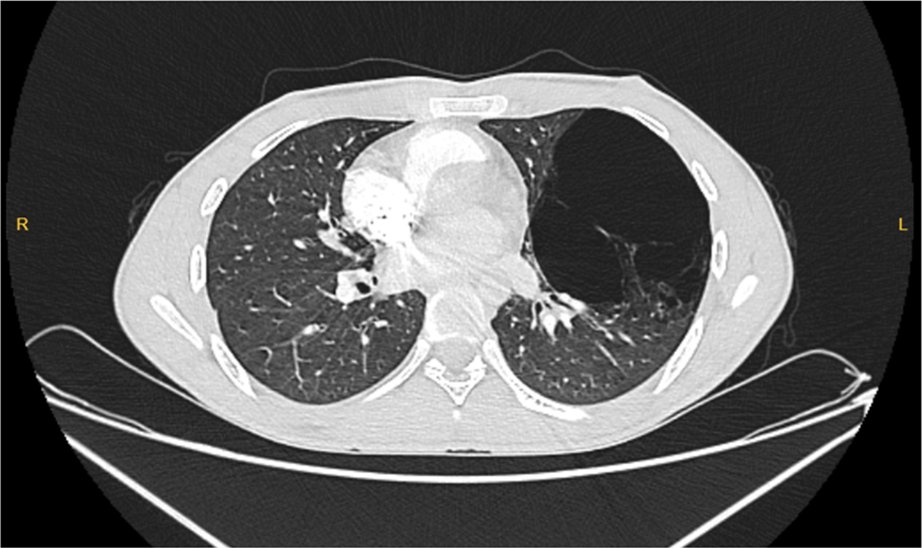
	Multiloculated areas demonstrating necrosis and internal air foci; mediastinal shift is present	A cystic lesion with thin internal septations is seen, surrounded by smaller cysts.
Oncological follow-up (duration/current status)	Clinical review every 3 months with annual chest MRI; No evidence of recurrence.	Clinical review every 3 months with annual chest MRI or CT; Transitioned to adult care; currently under follow-up by medical oncology and pulmonology. No evidence of recurrence.
Current age and follow-up period	33 months (33 **months)**	20 years (5 **years)**

CPAM, congenital pulmonary airway malformation; CT, computed tomography; KRAS, Kirsten rat sarcoma viral oncogene homologue; MRI, magnetic resonance imaging; Op, operation.

## Discussion

This study presents several findings of direct clinical significance beyond confirming that CTMs undergoing surgery are consistent with normal somatic growth and near-normal lung function. First, an 80.40% agreement between preoperative radiology and postoperative histopathology findings supports the reliability of multidisciplinary, CT-based preoperative assessment for surgical planning. Second, the observation of significantly higher weight z-scores (*p* = 0.004) after VATS compared to thoracotomy, even though both groups remained within normal reference ranges, raises the hypothesis that a minimally invasive approach may better preserve long-term nutritional and growth trends; a question that our retrospective data with confounding factors may raise but not answer. Third, the shift toward VATS as the dominant approach in 2024–2025 (1 thoracotomy versus 13 VATS procedures) reflects the international trend toward thoracoscopic resection and demonstrates the institution's accumulated experience. Combined with a detailed pediatric pulmonology follow-up datasets from a tertiary care cohort, these observations translate into two practical messages: the surgical approach should be personalized to the child's clinical condition and the center's expertise; and structured, multidisciplinary pulmonology follow-up is essential to confirm the generally positive outcome these children experience.

The concordance rate of the radiologic and pathologic diagnoses was 80.40%, which falls within the upper limits of the range reported in the literature (65.4%–84%) (3, 9, 10). This high rate may be attributable to factors such as the center being a tertiary referral hospital with a multidisciplinary approach, optimized CT scanning timing, the use of advanced imaging techniques and high-end equipment, and the expertise of the pediatric radiology team.

In this study, two patients were diagnosed with CPAM and mucinous adenocarcinoma, with one exhibiting a KRAS mutation. While overt malignant transformation of CTMs is extremely rare, there are reports of pleuropulmonary blastoma/rhabdomyosarcoma arising in CPAM lesions (particularly type 4) (11, 12). Cases of mucinous adenocarcinoma developing on a CPAM background have also been reported (12). In a study by Chang W et al., the pathology series included 37 cases of mucinous adenocarcinoma associated with CPAM. Of these, 33 were associated with CPAM type 1 and 4 with CPAM type 2, indicating a predominance of type 1 lesions in the development of mucinous adenocarcinoma (13). Van Horrik C. et al. reported that KRAS mutations may be a risk factor for adenocarcinoma development in the context of CPAM (14). This underscores the importance of careful histopathological evaluation of resected CPAM samples and suggests that molecular evaluation (e.g., KRAS testing) may be informative, particularly in selected cases with mucinous proliferation. Further multicenter studies with systematic molecular profiling are needed to better define the incidence, subtype-specific risk, and clinical utility of genetic markers for risk stratification. Congenital lung malformations (especially CPAM, sequestration, and BCs), although rare, carry a risk of malignant transformation. It is generally accepted that surgical resection significantly reduces but does not eliminate this risk (15, 16). In the series reported by Muntean et al., 210 patients underwent surgical resection for CPAM, of whom 43 were in the neonatal period. Among these, three were found to harbor mucinous adenocarcinoma with a positive KRAS mutation and were managed with follow-up alone, without adjuvant chemotherapy (17). Malignant transformation is a well-recognized complication of CPAM, and reported cases span a wide age range, from neonates to adults. Our series is notable in encompassing both ends of this spectrum, including one neonatal patient and one older patient. The neonatal presentation parallels the series by Muntean et al., which demonstrated that mucinous adenocarcinoma may already be present in the newborn period, whereas our older patient is consistent with the more commonly reported pattern of malignant transformation occurring later in childhood or adulthood. Across these cases, the association with Stocker type 1 lesions and somatic KRAS mutations is a recurring finding. The occurrence of malignancy at both extremes of age in our experience underscores that the malignant potential of CPAM persists throughout life and supports elective resection together with thorough histopathological sampling of all excised specimens, regardless of the patient's age at presentation. CTMs should be monitored closely as they can mask tumors, even if surgically resected.

Prenatal diagnosis was established in 34 cases (60.70%), consistent with studies reporting accuracies of 35%–74% (18, 19). The high prenatal diagnosis rate observed in this study may be attributable to the clinic being a tertiary referral center with a multidisciplinary approach.

The median age at operation was 12.50 months, as in the literature (20). Patients diagnosed prenatally underwent surgery earlier, although this trend was not statistically significant. Similarly, when patients were examined according to the timing of surgery, there was a trend toward a decrease in the age at operation in the last 2 years of the study, although this was not statistically significant. The optimal timing for surgery for asymptomatic CTMs at birth is still debated (21). A survey of pediatric surgeons from 48 countries found that 62% of asymptomatic CPAM surgeries were performed on infants aged 6–12 months (4). However, several previous studies recommend early surgical intervention at 1–6 months to prevent infection and promote compensatory lung growth (22). Uppala et al. found that early surgery was preferable in asymptomatic children (48.6% asymptomatic), with the complication rate lower than in symptomatic children (*p* = 0.028) (23). In recent years, patients diagnosed prenatally have begun to undergo surgery earlier (23).

A systematic review and meta-analysis by Liu et al. indicated that thoracoscopic lobectomy can reduce overall complications, particularly respiratory complications, but does not offer a consistent advantage in terms of hospital stay duration (24). Recent studies indicate that VATS is associated with significantly shorter hospital stays (20, 25). While the current study found that the length of hospitalization was shorter in patients who underwent VATS (10 days) than those who underwent thoracotomy (14 days), this difference was not statistically significant. This may be attributable to the retrospective design and relatively small sample size. While shorter hospital stays favoring VATS are reported in the literature, the statistical significance of this difference remains uncertain (20, 24, 25). This difference is thought to be due to patient selection, differences in surgical and clinical practices between centers, and heterogeneity in preoperative and postoperative care protocols (20, 24, 25).

The current study found a statistically significant difference in age at operation between patients who underwent thoracotomy and those who underwent VATS (4 vs. 19 months), with thoracotomy typically performed at younger ages. A study by Kunisaki S. M et al., which included only patients aged < 10 years and weighing < 25 kg, reported a difference in age between VATS (1.5 years) and thoracotomy (0.9 years); however, this was not statistically significant (26). A study by Lan T. Vu et al. reported the mean patient ages as 6.7 months for VATS and 3.7 months for thoracotomy, with the between-group difference also nonsignificant (27). In our cohort, thoracotomy was performed predominantly in neonates and young infants who required emergency surgery for acute respiratory failure, whereas VATS was reserved for elective operations in older, asymptomatic children. Accordingly, the open approach predominated among patients who were diagnosed postnatally and symptomatic at presentation. This pattern is further supported by the finding that 5 of the 13 open procedures (38.50%) were intraoperative conversions from VATS, indicating that the decision to proceed with an open approach was, in some cases, dictated by intraoperative findings rather than made preoperatively. Conversion to open thoracotomy was required in five patients due to inadequate surgical visualization during VATS. Such differences in reported results may originate from variations in study methodology, the periods during which the surgeries were performed, and the centers' experience levels (26, 27).

Several studies have shown that the risk of overt conversion (in the surgery) increases with active infection and a history of previous infection (27, 28). However, it should be noted that the VATS approach has been increasingly adopted in recent years, regardless of patient age (28). Consistent with this trend, most surgeries performed in the last two years of this study were VATS; thoracotomy was preferred in only one case. The rate of conversion to intraoperative thoracotomy was high in cases initiated with VATS. This rate ranges from 18.0% to 20.6% in the literature (20, 29) and is thought to be associated with case complexity and surgeon experience.

Regarding respiratory morbidity, this cohort showed a low incidence of chronic pulmonary symptoms. Only 8.9% experienced recurrent or refractory pneumonia, with 16.1% diagnosed with asthma by a physician during follow-up. Wheezing was uncommon. This indicates that long-term respiratory symptoms are rare following CTM resection (2, 30).

Patient height and weight z-scores were within normal limits at the final follow-up visit; consistent with the literature, there was no difference between patients with and without a prenatal diagnosis (31). No significant differences were observed between the height and weight z-scores of patients diagnosed pre- and postnatally; both groups were within the normal range. On the other hand, a noteworthy finding emerged from the evaluation based on surgical technique (VATS vs. thoracotomy). A significant difference in groups was observed in weight z-scores, with higher values in the VATS group, although both groups remained within age-appropriate normal limits. Because thoracotomy was preferentially performed in younger neonates operated for acute respiratory failure while VATS was used electively in older, often asymptomatic children, this difference is likely confounded by indication and by age at surgery and should be regarded as hypothesis-generating rather than as evidence that the VATS approach itself improves growth. This potential association warrants validation in prospective, adequately powered studies that adjust for baseline clinical severity.

No significant differences were observed between VATS and thoracotomy with respect to scoliosis, chest deformity, and spirometric parameters during long-term follow-up, consistent with Lam et al. (32). Spirometric values were within normal limits in the study by Todo M. et al., but lower than those of the healthy group (33).

Another finding was the frequency of postoperative musculoskeletal sequelae. At the final follow-up, 17.9% of patients had developed scoliosis and 14.3% had developed pectus excavatum or carinatum, consistent with the literature (30, 34). In our cohort, all scoliosis and pectus (excavatum/carinatum) deformities detected during follow-up were clinically mild, and none of the patients required surgical intervention or the use of a medical device. These children continue to be monitored at three-month intervals within a multidisciplinary follow-up program involving pediatric surgery, pediatric pulmonology, neurosurgery, and physical medicine and rehabilitation. These findings agree with earlier reports that most pediatric patients who underwent lobectomy for congenital lung lesions had lung function nearly back to normal (35). The prospective study by Naito et al. found no association between age at lobectomy (newborns to approximately 4.5 years) and subsequent lung function or exercise capacity (35). A 2023 systematic review of 10 studies found that among children who underwent lung resection, the mean FEV₁ was 80% of the predicted value, the FVC was 88% of the predicted value, and the combined FEV₁/FVC ratio was 94% (approximate values) (36). Within the limited spirometry subgroup (*n* = 15), we did not detect a statistically significant difference in lung function between diagnostic or surgical groups. Given the small subgroup and the resulting low power, these comparisons are exploratory and cannot exclude clinically meaningful differences. This suggests that lung capacity recovers regardless of whether surgery is performed in infancy or several years later (24, 33, 36).

From a patient and family-centered perspective, the value of this data lies not only in the numbers but also in what clinicians can offer families: a sense of reassurance based on evidence. For parents facing a prenatal diagnosis of congenital thoracic malformation which often occurs during an already anxious pregnancy, being able to communicate that the vast majority of children who undergo surgery grow normally, maintain lung function, and experience very few chronic respiratory complications is, in itself, a meaningful therapeutic action. Therefore, we advocate for a follow-up model that addresses the child holistically, rather than focusing solely on the resected lobe: long-term monitoring of growth and development, attention to musculoskeletal sequelae such as scoliosis and chest wall deformities, age-appropriate spirometry when the child is developmentally ready, and providing families with clear and empathetic counseling about both reassuring long-term prognosis and the remaining risks, albeit rare, such as malignant transformation. Integrating this humane, family-centered approach into the standard follow-up process is, in our view, as important as the surgical decision itself.

The present cohort was not designed to determine the optimal timing of surgery, and the observed difference in age at operation between prenatally and postnatally diagnosed patients was not statistically significant; our data therefore cannot support a recommendation on timing. On the basis of the existing literature rather than of the present findings, one reasonable approach in asymptomatic congenital thoracic malformations is detailed anatomical evaluation of the lesion by thoracic computed tomography in the second month of life, with elective resection at approximately the fourth month in patients deemed suitable for surgical resection (1). This approach is thought to allow surgical treatment to be completed before the development of infection and to take advantage of compensatory lung growth in the early period. However, it should be borne in mind that there is no definitive consensus regarding the optimal timing of surgery. In the postoperative period, we propose a standardized, multidisciplinary pulmonology follow-up protocol including serial anthropometric assessment, structured respiratory symptom review, spirometry from the time the child can reliably cooperate (usually around 6 years of age), periodic screening for musculoskeletal sequelae, and given the two mucinous adenocarcinoma cases in this series, continuous vigilance for latent malignant transformation with a low threshold for cross-sectional imaging.

### Limitations

This study has several limitations. First, the retrospective and single-center design limits the generalizability of the study due to differences in surgical and follow-up practices between centers. Second, respiratory function data were available for only 15 (26.70%) of the 56 patients; this reflects the young age of a large proportion of the cohort (median age at last visit 54 months) and the developmental maturity required for spirometry; therefore, spirometry comparisons are exploratory and subject to sampling bias; furthermore, the lack of a significant difference between prenatal and postnatal should be interpreted with caution. Third, owing to the retrospective, single-center design, the sample size was determined by case availability rather than by an *a priori* power calculation, and the study remained underpowered for small to moderate effects. For the comparison between the VATS (*n* = 43) and thoracotomy (*n* = 13) groups, the available sample provided 69.9% power to detect a large between-group difference (d = 0.8), but only 34.2% power to detect a moderate difference (d = 0.5); sensitivity analysis indicated that the smallest difference detectable with 80% power was d = 0.90. For the comparison between prenatally (*n* = 34) and postnatally (*n* = 22) diagnosed patients, the corresponding values were 81.9% power for a large effect, 43.5% power for a moderate effect, and a minimum detectable effect size of d = 0.78. Detecting a moderate difference with 80% power would have required approximately 179 patients given the observed 3.3:1 group allocation ratio, or approximately 128 patients if the groups had been balanced. Because pulmonary function tests could be performed in only 15 patients (26.70%), power for spirometric comparisons was substantially lower; only very large differences (d > 1.5) would have been detectable in this subgroup. Categorical outcomes were similarly limited: at the observed frequencies, an absolute difference of approximately 33 percentage points between the prenatally and postnatally diagnosed groups would have been required to achieve 80% power, whereas the observed differences in scoliosis (11.80% vs. 27.30%) and pectus deformity (11.80% vs. 18.20%) corresponded to statistical power of 32.30% and 11.10%, respectively. Accordingly, the non-significant comparisons reported here should be interpreted as the absence of detectable large effects rather than as evidence of equivalence between-groups; clinically meaningful smaller differences cannot be excluded. In contrast, the adjusted between-group difference in weight z-score corresponded to a large effect (d ≈ 0.96), which is within the range detectable with the available sample. Fourth, the two surgical groups differed systematically in age and clinical presentation. To address this, the primary between-group outcome (weight z-score at the last visit) was adjusted for age at operation and duration of follow-up using analysis of covariance, and the effect of surgical approach persisted as a large, statistically significant difference (adjusted d ≈ 0.96), which was within the range detectable with the available sample. This adjustment cannot, however, account for unmeasured confounders most importantly baseline disease severity because thoracotomy was preferentially performed in younger, acutely symptomatic neonates; this association should therefore be regarded as hypothesis-generating rather than causal. The remaining VATS versus thoracotomy comparisons (spirometry, length of hospitalization, and musculoskeletal outcomes) were not adjusted and are similarly subject to confounding by clinical indication. Fifth, the oncological follow-up after the two cases of mucinous adenocarcinoma is short, precluding definitive conclusions about long-term malignancy outcomes. Finally, the median follow-up of approximately 20.50 months is relatively short for long-term outcome assessment, and operative duration could not be retrieved retrospectively.

## Conclusion

In this single-center retrospective cohort, surgical resection of CTMs in infancy or early childhood was associated with favorable long-term outcomes: somatic growth was normal, postoperative pulmonary function was near-normal in the subset assessed by spirometry (*n* = 15), and recurrent respiratory morbidity was low. Prenatal diagnosis allowed earlier evaluation and treatment planning, and the choice between VATS and thoracotomy was driven largely by clinical presentation and center experience. Because patients were not randomly allocated and the two surgical groups differed systematically in age and symptom status, these data cannot establish the optimal timing of surgery or demonstrate the superiority of either surgical approach; the higher weight z-scores observed after VATS are confounded by indication and by age at operation and should be regarded as hypothesis-generating. Within these limitations, close monitoring of growth and development during long-term follow-up appears important, with early nutritional support if growth falters. These observations may help clinicians counsel families about expected postoperative outcomes, and require confirmation in larger, prospective, multicenter studies.

## Data Availability

The original contributions presented in the study are included in the article/Supplementary Material, further inquiries can be directed to the corresponding author.

## References

[1] Pederiva F Rothenberg SS Hall N Ijsselstijn H Wong KKY Von Der Thüsen J. Congenital lung malformations. Nat Rev Dis Primers. (2023) 9(1):60. 10.1038/s41572-023-00470-1 37919294

[2] Nuutinen S Ronkainen E Perhomaa M Harju T Sinikumpu JJ Serlo W. Long-Term results of pediatric congenital pulmonary malformation: a population-based matched case–control study with a mean 7-year follow-up. Children. (2023) 10(1):71. 10.3390/children10010071 PMC9857330 36670622

[3] Farrugia MK Raza SA Gould S Lakhoo K. Congenital lung lesions: classification and concordance of radiological appearance and surgical pathology. Pediatr Surg Int. (2008) 24(9):987–91. 10.1007/s00383-008-2201-1 18665370

[4] Morini F Zani A Conforti A Van Heurn E Eaton S Puri P. Current management of congenital pulmonary airway malformations: a “European pediatric Surgeons' Association” survey. Eur J Pediatr Surg. (2018) 28(1):1–5. 10.1055/s-0037-1604020 28709163

[5] Miller MR Hankinson J Brusasco V Burgos F Casaburi R Coates A. Standardisation of spirometry. Eur Respir J. (2005) 26(2):319–38. 10.1183/09031936.05.00034805 16055882

[6] Quanjer PH Stanojevic S Cole TJ Baur X Hall GL Culver BH. Multi-ethnic reference values for spirometry for the 3–95-yr age range: the global lung function 2012 equations. Eur Respir J. (2012) 40(6):1324–43. 10.1183/09031936.00080312 22743675 PMC3786581

[7] Graham BL Steenbruggen I Miller MR Barjaktarevic IZ Cooper BG Hall GL. Standardization of spirometry 2019 update. An official American thoracic society and European respiratory society technical statement. Am J Respir Crit Care Med. (2019) 200(8):e70–88. 10.1164/rccm.201908-1590ST 31613151 PMC6794117

[8] Faul F Erdfelder E Lang AG Buchner A. G*power 3: a flexible statistical power analysis program for the social, behavioral, and biomedical sciences. Behav Res Methods. (2007) 39(2):175–91. 10.3758/BF03193146 17695343

[9] Lanza C Bolli V Galeazzi V Fabrizzi B Fabrizzi G. Cystic adenomatoid malformation in children: cT histopathological correlation. Radiol Med. (2007) 112(4):612–9. 10.1007/s11547-007-0166-0 17563845

[10] Mon RA Johnson KN Ladino-Torres M Heider A Mychaliska GB Treadwell MC. Diagnostic accuracy of imaging studies in congenital lung malformations. Arch Dis Child Fetal Neonatal Ed. (2019) 104(4):F372–f7. 10.1136/archdischild-2018-314979 30049725

[11] Nasr A Himidan S Pastor AC Taylor G Kim PCW. Is congenital cystic adenomatoid malformation a premalignant lesion for pleuropulmonary blastoma? J Pediatr Surg. (2010) 45(6):1086–9. 10.1016/j.jpedsurg.2010.02.067 20620300

[12] Casagrande A Pederiva F. Association between congenital lung malformations and lung tumors in children and adults: a systematic review. J Thorac Oncol. (2016) 11(11):1837–45. 10.1016/j.jtho.2016.06.023 27423390

[13] Chang WC Zhang YZ Wolf JL Hermelijn SM Schnater JM Von Der Thüsen JH. Mucinous adenocarcinoma arising in congenital pulmonary airway malformation: clinicopathological analysis of 37 cases. Histopathology. (2021) 78(3):434–44. 10.1111/his.14239 32810914

[14] van Horik C Benthem F Buscop-van Kempen M Boerema-de Munck A Van Ijcken WFJ Wijnen RMH. Activating KRAS mutations mark premalignant cystic structures in congenital pulmonary airway malformations. Respir Res. (2026) 27(1):56. 10.1186/s12931-025-03491-4 41540433 PMC12892516

[15] Papagiannopoulos KA Sheppard M Bush AP Goldstraw P. Pleuropulmonary blastoma: is prophylactic resection of congenital lung cysts effective? Ann Thorac Surg. (2001) 72(2):604–5. 10.1016/S0003-4975(00)02539-X 11515907

[16] Benjamin DR Cahill JL. Bronchioloalveolar carcinoma of the lung and congenital cystic adenomatoid malformation. Am J Clin Pathol. (1991) 95(6):889–92. 10.1093/ajcp/95.6.889 1645926

[17] Muntean A Banias LE Ade-Ajayi N Patel SB McKinney O Davenport M. Neonatal congenital pulmonary airway malformation associated with mucinous adenocarcinoma and KRAS mutations. J Pediatr Surg. (2022) 57(11):520–6. 10.1016/j.jpedsurg.2021.12.018 34980466

[18] Alamo L Reinberg O Vial Y Gudinchet F Meuli R. Comparison of foetal US and MRI in the characterisation of congenital lung anomalies. Eur J Radiol. (2013) 82(12):e860–6. 10.1016/j.ejrad.2013.09.012 24119429

[19] Puumalainen T Kauppinen T Nikkinen H. Prenatal diagnostic accuracy and epidemiology of congenital lung malformations: a retrospective review of cases in a tertiary referral center in northern Finland in 2010–2020. Acta Obstet Gynecol Scand. (2025) 104(6):1120–7. 10.1111/aogs.15100 40325854 PMC12087522

[20] Ganarin A Sgrò A Garcia Magne M Volpe A Tognon C Gamba P. Thoracoscopy versus thoracotomy for congenital lung malformations treatment: a single center experience. Pediatr Pulmonol. (2021) 56(1):196–202. 10.1002/ppul.25138 33111504

[21] Duron V Zenilman A Griggs C Defazio J Price J Fan W. Asymptomatic congenital lung malformations: timing of resection does not affect adverse surgical outcomes. Front Pediatr. (2020) 8:35. 10.3389/fped.2020.00035 32117840 PMC7033465

[22] Laberge JM Puligandla P Flageole H. Asymptomatic congenital lung malformations. Semin Pediatr Surg. (2005) 14(1):16–33. 10.1053/j.sempedsurg.2004.10.022 15770585

[23] Uppala R Udomdirekkul P Niamsanit S Techasatian L Saengnipanthkul S Sitthikarnkha P. Clinical outcomes after surgical resection in asymptomatic and symptomatic children with congenital lung malformations. J Cardiothorac Surg. (2025) 20(1):126. 10.1186/s13019-025-03372-3 39955568 PMC11829428

[24] Liu X Wu Z Li X. Thoracoscopic versus thoracotomy lobectomy in children with congenital lung lesions: a systematic review and meta-analysis. ANZ J Surg. (2024) 94(1-2):208–14. 10.1111/ans.18859 38263509

[25] Clark RA Perez EA Chung DH Pandya SR. Predictive factors and outcomes for successful thoracoscopic lung resection in pediatric patients. J Am Coll Surg. (2021) 232(4):551–8. 10.1016/j.jamcollsurg.2020.12.013 33359619

[26] Kunisaki SM Powelson IA Haydar B Bowshier BC Jarboe MD Mychaliska GB. Thoracoscopic vs open lobectomy in infants and young children with congenital lung malformations. J Am Coll Surg. (2014) 218(2):261–70. 10.1016/j.jamcollsurg.2013.10.010 24315887

[27] Vu LT Farmer DL Nobuhara KK Miniati D Lee H. Thoracoscopic versus open resection for congenital cystic adenomatoid malformations of the lung. J Pediatr Surg. (2008) 43(1):35–9. 10.1016/j.jpedsurg.2007.09.012 18206452

[28] Polites SF Habermann EB Zarroug AE Thomsen KM Potter DD. Thoracoscopic vs open resection of congenital cystic lung disease- utilization and outcomes in 1120 children in the United States. J Pediatr Surg. (2016) 51(7):1101–5. 10.1016/j.jpedsurg.2015.12.004 26794289

[29] Weller JH Peter SDS Fallat ME Saito JM Burns CR Deans KJ. Thoracoscopic versus open lobectomy in infants with congenital lung malformations: a multi-institutional propensity score analysis. J Pediatr Surg. (2021) 56(12):2148–56. 10.1016/j.jpedsurg.2021.04.013 34030879

[30] Calzolari F Braguglia A Valfrè L Dotta A Bagolan P Morini F. Outcome of infants operated on for congenital pulmonary malformations. Pediatr Pulmonol. (2016) 51(12):1367–72. 10.1002/ppul.23472 27232731

[31] Ramaslı Gürsoy T Ramaslı Gürsoy T Aslan AT Karabulut R Taştepe A. Evaluation of children with congenital lung malformations who were diagnosed in the prenatal and postnatal period. Turk J Pediatr Dis. (2022) 16(4):336–42. 10.12956/tchd.1034487

[32] Lam FKF Lau CT Yu MO Wong KKY. Comparison of thoracoscopy vs. Thoracotomy on musculoskeletal outcomes of children with congenital pulmonary airway malformation (CPAM). J Pediatr Surg. (2021) 56(10):1732–6. 10.1016/j.jpedsurg.2021.01.028 33551147

[33] Todo M Deguchi K Okuyama H Watanabe M. Long-term outcomes of children undergoing thoracotomy lung resection for congenital lung malformations. Surg Today. (2026) 56:1461–9. 10.1007/s00595-026-03242-y 41670670 PMC13379478

[34] Beres A Aspirot A Paris C Berube D Bouchard S Laberge JM. A contemporary evaluation of pulmonary function in children undergoing lung resection in infancy. J Pediatr Surg. (2011) 46(5):829–32. 10.1016/j.jpedsurg.2011.02.012 21616235

[35] Naito Y Beres A Lapidus-Krol E Ratjen F Langer JC. Does earlier lobectomy result in better long-term pulmonary function in children with congenital lung anomalies? A prospective study. J Pediatr Surg. (2012) 47(5):852–6. 10.1016/j.jpedsurg.2012.01.037 22595560

[36] Liu C Liu J Yuan M Cheng K Luo D Zeng L. Pulmonary function after lobectomy in children: a systematic review and meta-analysis. BMJ Paediatr Open. (2023) 7(1):e001979. 10.1136/bmjpo-2023-001979 37848263 PMC10582896

